# Donor cornea transfer from Optisol GS to organ culture storage: a two-step procedure to increase donor tissue lifespan

**DOI:** 10.1111/j.1755-3768.2012.02390.x

**Published:** 2013-05

**Authors:** Kristiane Haug, Amaya Azqueta, Siv Johnsen-Soriano, Aboulghassem Shahdadfar, Liv K Drolsum, Morten C Moe, Magnus T Røger, Francisco J Romero, Andrew R Collins, Bjørn Nicolaissen

**Affiliations:** 1 Center for Eye Research, Department of Ophthalmology, Oslo University Hospital, Ullevål and University of OsloOslo, Norway; 2 Department of Nutrition, Institute for Basic Medical Sciences, University of OsloOslo, Norway; 3 Fundación Oftalmológica del MediterránoValencia, Spain; 4 Department of Pathology, Oslo University Hospital, Ullevål and University of OsloOslo, Norway; 5 Universidad CEU Cardenal HerreraValencia, Spain

**Keywords:** cell damage, cornea, differentiation, epithelium, limbus, Optisol GS, organ culture, oxidative damage, stem cells

## Abstract

**Purpose:**

Storage time for donor corneas in Optisol GS is limited compared to Eye Bank Organ Culture (EBOC). We here examine the epithelium on donor corneoscleral rims after primary storage in Optisol GS and subsequent incubation in EBOC.

**Methods:**

Morphology was monitored by light and electron microscopy, expression of phenotypic and genotypic markers by immunohistochemistry and RT-PCR and changes in oxidative lipid and DNA damage by ELISA and COMET assay.

**Results:**

A prominent loss of cells was observed after storage in Optisol GS. After maintenance in EBOC, spreading apical cells were Occludin^+^, while the staining for E-cadherin and Connexin-43 was less intense. There were an upregulation of *Occludin* and a downregulation of *E-cadherin* and *Connexin-43*. Eye Bank Organ Culture was associated with an ongoing proliferative activity and a downregulation of putative progenitor/stem cell marker *ABCG2* and *p63*. Staining for 8-OHdG and Caspase-3 did not increase, while levels of malondialdehyde and number of DNA strand breaks and oxidized bases increased.

**Conclusions:**

This dual procedure should be pursued as an option to increase the storage time and the pool of available donor corneas. The observed downregulation of markers associated with stemness during EBOC is relevant considering the potential use of donor epithelium in the treatment of ocular surface disorders.

## Introduction

‘Cold’ storage at 4°C and organ culture at 32°C are two methods commonly used in order to prolong donor corneal survival, both with similar results as for long-term postoperative outcome (Rijneveld et al. 2008). Prevailing method in the United States is cold storage (CS), and the upper limit for the use of the donor tissue is 14 days. Changes in the epithelium include progressive detachment of epithelial layers, cell death and formation of defects with exposure of the basement membrane (Greenbaum et al. 2004).

In European countries, the commonly used procedure is Eye Bank Organ Culture (EBOC) (Kolstad 1979; Armitage & Easty 1997; Hjortdal et al. 1997; Borderie et al. 2006; Rijneveld et al. 2008; Ehlers et al. 2009). This system permits the maintenance of tissue for up to 4 weeks, and epithelial defects present prior to preservation may to some extent heal during the first 3 days in an organ culture system (Slettedal et al. 2007).

During storage and also during transport and international exchange of donor corneas, the preservation time for tissue in Optisol GS may approximate the upper recommended limit, and transfer to EBOC has been suggested as an option to increase the lifespan of donor corneas (Nelson et al. 1984; Camposampiero et al. 2003; Rijneveld et al. 2011). Such a dual approach may increase the pool of available donor tissue (Rijneveld et al. 2011) and may, in addition, permit some regeneration of epithelial defects prior to release of tissue for surgery.

In the present study, we used human corneoscleral rims retrieved after transplant procedures and where the donor corneas had been maintained in Optisol GS prior to surgery. Several previous studies have provided a considerable amount of information about the behaviour of the epithelium, the stroma and the endothelium during Eye Bank storage in Optisol GS and in organ culture. For transfer and prolonged storage of donor tissue, each of these storage methods may serve as a second procedure. We here examined the epithelium for changes in architecture, ultrastructure, proliferation and expression of selected phenotypic and genotypic markers associated with a secondary incubation in a commonly used EBOC system. Considering previous reports on cellular stress during Eye Bank storage (Komuro et al. 1999; Jeng et al. 2002), we also monitored epithelial staining for 8-OHdG, Caspase-3 and TUNEL and the levels of lipid peroxides and DNA damage profiles.

## Materials and Methods

### Tissue

Corneas were stored in Optisol GS (Bausch & Lomb Incorporated, Rochester, NY, USA) at 4°C until transplantation, and the remaining corneoscleral rim acquired for our study. Half of the rims (*n* = 20) were immediately processed for analysis (Optisol GS, group 1), while the other half (*n* = 20) was transferred to Eye Bank OC for 1 week prior to analysis (Optisol GS + OC, group 2). This experimental design was selected in order to examine the effect of OC on tissue previously stored in Optisol GS. In studies on DNA damage, additional 10 rims were used. Mean donor age in group 1 was 61.5 (SD 10.1) years and in group 2, 64.8 (SD 8.8) years; post-mortem time to preservation in the two groups was 9.9 (SD 4.0)/10.5 (SD 3.9) hr, time from preservation to transplantation was 9.8 (SD 1.3) days, and time in OC was 6.0 (SD 0.7) days. Female/male ratio was 11:29. In the group for DNA damage, age was 57 (SD 13.4) years, post-mortem time was 7 (SD 5.9) h, storage in Optisol GS was 8.7 (SD 1.5) days, and time in organ culture was 6.7 (SD 0.8) days. The study was approved by the Regional Committee for Medical Research Ethics of Eastern Norway, all tissue was consented for research, and the Helsinki Declaration was adhered to throughout the study.

### Eye Bank storage systems

Cold storage medium at 4°C: Optisol GS (Bausch & Lomb Incorporated, Rochester, NY, USA) was used in CS medium. Eye Bank Organ Culture medium at 32°C: Eagle’s MEM with Earle salts and l-glutamate (Gibco, Invitrogen, Paisley, UK), sodium bicarbonate (2.20 μg/ml), HEPES buffer (2.98 μg/ml), 8% heat-inactivated foetal calf serum, amphotericin B (5 μg/ml), gentamicin (50 μg/ml) (Sigma Aldrich, Saint Louis, MO, USA) and Vancomycin (100 μg/ml) (Alpharma ApS, Copenhagen, Denmark), pH 7.1–7.2, was used in EBOC medium.

### Light microscopy, immunostaining and ultrastructure

From each rim, duplicate or triplicate samples were removed, fixed in 4% formaldehyde and embedded in paraffin; 3-μm sections were stained with haematoxylin and eosin (H&E), and nuclei of the epithelium were counted and their appearance was recorded. Sections were also stained for Ki-67 (SP6, 1:200; Thermo Scientific, Freemont, CA, USA), PCNA (PC10, 1:1500; DakoCytomation, Glostrup, Denmark), p63 (4A4 + Y4A3, 1:1600; Thermo Scientific), p63α (C-12, 1:1500; Santa Cruz Biotechnology, Santa Cruz, CA, USA), ABCG2 (BXP-21, 1:80; Sigma Aldrich, Saint Louis, MO, USA), E-cadherin (NCH-38, 1:50; DakoCytomation), Vimentin (SP20, 1:200; Thermo Scientific, Freemont, CA, USA), Occludin (ab64482, 1:70; AbCam, Cambridge, UK), 8-OHdG (15A3, 1:100; Santa Cruz Biotechnology, Santa Cruz, CA, USA), Connexin-43 (C6219, 1:500; Sigma Aldrich, Saint Louis, MO, USA) and Caspase 3 (ASP175 5A1, 1:200; Cell Signaling, Beverly, MA, USA). Cytokeratin 3 (AE3, 1:500; ImmuQuest, Cleveland, UK) was used to confirm a corneal epithelial phenotype. An Autostainer360 (Lab Vision Corporation, Fremont, CA, USA) was used, and positive immunoreactions were detected by a secondary antibody conjugated with peroxidase-labelled polymer with diaminobenzidine. Positive and negative controls were used as recommended by distributor. Total cell number, Ki-67^+^, PCNA^+^ and p63^+^ cells were counted at 40× magnification by three independent observers in the peripheral corneal epithelium. Student’s *t*-test and Statistical Package for the Social Sciences (spss version 18; IBM Corporation, Armonk, NY, USA) were used. In the limbal area, the number of Ki67^+^ and ABCG2^+^ cells and their distribution in basal versus suprabasal layers were recorded (triplicate counts at 40× magnification, see Results). For 8-OHdG and Caspase-3, the reaction was evaluated in sections from six random samples maintained in Optisol GS or in Optisol GS + organ culture.

Terminal deoxyribonucleotidyl transferase–mediated dUTP nick-end DNA labelling (TUNEL) was used to detect apoptotic cells (DeadEnd™ Colorimetric TUNEL System; Promega, Madison, WI, USA).

For transmission electron microscopy (TEM), samples were fixed in 2% glutaraldehyde in 0.2 m cacodylate buffer, washed, postfixed in osmium tetroxide, dehydrated and embedded in Epon. Ultrathin sections (60–70 nm) were cut on a Leica Ultracut Ultramicrotome UCT (Leica, Wetzlar, Germany), contrasted with uranyl acetate and lead citrate and examined in a transmission electron microscope (Tecnai G2 Spirit BioTWIN 120 kV, LaB6; FEI Company, Eindhoven, the Netherlands).

### Real-time RT-PCR

RNA was extracted using Qiazol reagent (Qiagen, Hilden, Germany). Following DNase treatment (Ambion, Austin, TX, USA), RNA was quantified by spectrophotometer (Nanodrop, Wilmington, DE, USA). Reverse transcription (RT) was performed using the High Capacity cDNA Archive Kit (Applied Biosystems, Abingdon, UK) with 200 ng of total RNA per 20 μl RT reaction. Comparative relative quantification was performed using the StepOnePlus Real-Time RT PCR system (Applied Biosystems) and Taqman® Gene Expression assays following protocols from the manufacturer (Applied Biosystems). All samples were run in triplicate. Data were analysed by the 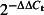 method for genes as the fold-change in expression, normalized to *GAPDH* as endogenous reference and expressed relative to Optisol GS, which was arbitrarily chosen as calibrator (Table 1).

**Table 1:** Primes used for real-time RT-PCR

Gene name	Gene symbol	Alias	Taqmen assay ID
ATP-binding cassette subfamily G2	*ABCG2*	*BCRP*	HS01053790_ml
Gap junction protein alpha 1, 43 kDa	*GJA1*	*CX43*	HS00748445_sl
Occludin	*OCLN*	–	HS00170162_ml
Glyceraldehyde-3-phosphate dehydrogenase	*GAPDH*	*GAPD*	HS99999905_ml
Tumour protein p63	*TP63*	*p63*	HS00978340_ml
Antigen identified by monoclonal antibody Ki67	*KI-67*	*MKI67*	HS01032443_ml
E-cadherin (epithelial)	*E-cadherin*	*ECAD*	HS01023894_ml
Tumour protein p53	*TP53*	*p53*	HS00153349_ml

### Lipid peroxidation by-products

#### ELISA

Lipid peroxidation was measured using a commercial kit (Lipid Peroxidation Microplate Assay Kit; Oxford Biomedical Research, Rochester Hills, MI, USA) following the manufacturer’s instructions. Reactive oxygen species degrade polyunsaturated lipids, forming malondialdehyde (MDA). The assay is based on the reaction of two molecules of a chromogenic reagent, *N*-methyl-2-phenylindole, with one molecule of MDA at 45°C to yield a stable chromophore with a maximal absorbance at 586 nm. The amount of MDA can be monitored by reading the absorbance at 586 nm, which is proportional to its concentration.

### DNA damage

Epithelium was removed by scraping in medium (Optisol GS or EBOC) on ice. A single-cell suspension is obtained by gentle pipetting and by centrifuging at 200 ***g*** for 5 min at 4°C, and cells were resuspended in 30 μl of phosphate-buffered saline (PBS). The comet assay (single-cell gel electrophoresis) (Azqueta et al. 2009) was used to measure strand breaks, oxidized pyrimidines (by digestion with endonuclease III; endo III), oxidized purines [with formamidopyrimidine-DNA glycosylase (FPG)] and cyclobutane pyrimidine dimers (with T4 endonuclease V; T4EndoV). Under fluorescence microscopy, after electrophoresis, comet-like images are seen, where the comet tail represents broken DNA that is able to move in the direction of the anode. The percentage of DNA in the tail indicates the frequency of breaks. For each sample, 25 comets per gel, two gels per treatment, were scored using a Nikon Eclipse TS-100 fluorescence microscope with semiautomated image analysis system (Comet Assay IV; Perceptive Instruments, Suffolk, UK), and the median was calculated as an index of the frequency of DNA lesions. To calculate net enzyme-sensitive sites, comet scores from buffer-incubated gels were subtracted from the scores of gels incubated with the different enzymes.

Untreated lymphocytes were used as a negative control, and as positive control, lymphocytes from healthy volunteers treated on ice with 2 μm photosensitizer Ro 19-8022 plus visible light (500-W tungsten-halogen source at 33 cm) to induce 8-oxoGua. They were treated as the corneal epithelium cells but incubated only with enzyme buffer or FPG, respectively.

Statistical Package for the Social Sciences (spss) was used for statistical analysis. A p-value <0.05 was considered statistically significant.

## Results

### Optisol GS storage (group 1)

**Fig. 1:**
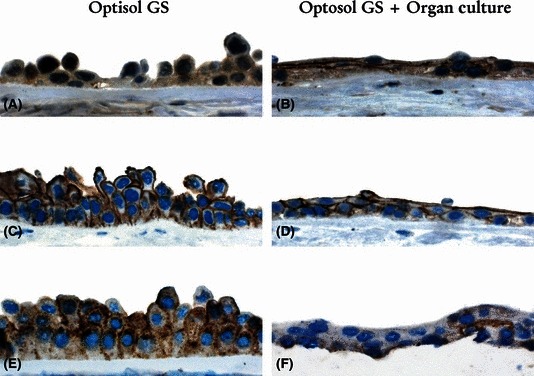
Immunostaining of adherens, tight and gap junction-associated markers Occludin (A, B), E-cadherin (C, D) and Connexin-43 (E, F) after storage in Optisol GS and after Optisol GS + EBOC. EBOC, Eye Bank Organ Culture.

**Fig. 2:**
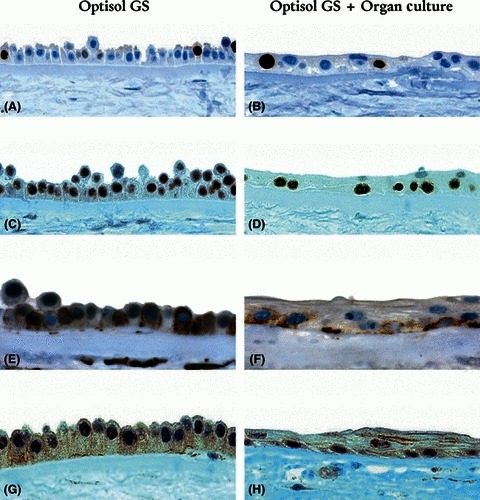
Immunostaining of proliferation-associated marker Ki-67 (A, B), stemness-associated marker p63α (C, D) Vimentin (E, F) and ABCG2 (G, H) of corneal epithelial cells after storage in Optisol GS and after Optisol GS + EBOC. Ki-67 and p63α are nuclear markers, and the positivity is shown by brown staining of the nucleus. Staining for Vimentin is prominent in cytoplasm, while positivity for ABCG2 is particularly evident along cell membranes. EBOC, Eye Bank Organ Culture.

Loss of superficial layers of the epithelium and additional detachment of scattered basal cells or groups of basal cells were observed on all rims. Most samples stained negative for the tight junction protein Occludin (Fig. 1) normally present between cells in the superficial layers, while most cells stained positive for E-cadherin and Connexin-43 (Fig. 1) markers for adherens and gap junctions, respectively. The proliferation marker Ki67 was positive in cells in the basal and in the suprabasal layer in the corneal epithelium (Fig. 2). Low-degree staining was also seen in the limbal area (4%), and the ratio between positive cells in the basal versus suprabasal layer was 0.3. Cells positive for the proliferation/progenitor/stem cell markers p63 and p63α were seen in the basal and suprabasal layer in the corneal as well as in the limbal epithelium. Vimentin stained basal limbal cells densely, while only few isolated suprabasal and some peripheral basal corneal cells were positive (Fig. 2). Similarly, dense staining for progenitor/stem cell marker ABCG2 was confined to cells in the basal limbal area, and staining of isolated suprabasal and corneal epithelial cells was less intense (Fig. 2).

**Fig. 3:**
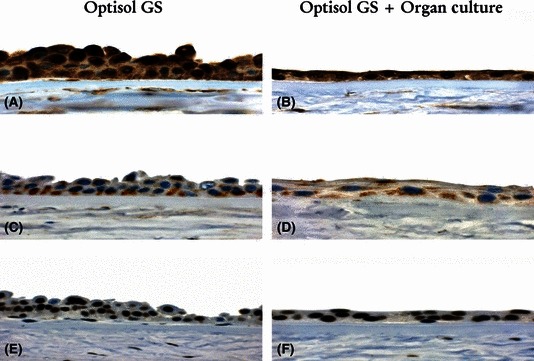
Immunostaining of DNA base oxidation and cell proliferation/DNA repair–associated markers 8-OHdG (A, B) and PCNA (E, F) and apoptosis marker Caspase 3 (C, D) after storage in Optisol GS and after Optisol GS + EBOC. EBOC, Eye Bank Organ Culture.

DNA base oxidation and cell proliferation/DNA repair as revealed by positive staining for 8-OHdG and PCNA, respectively, ranged between slight and intense. Staining for Caspase-3, a marker of apoptosis, was particularly evident in some cells with gross nuclear alterations, but a low to moderate activity was also seen in some cells with an apparent normal nuclear morphology (Fig. 3). The TUNEL assay was positive in some cells with deformed nuclei (picture not shown).

**Fig. 4:**
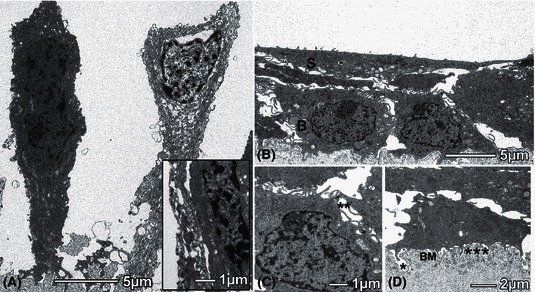
Transmission electron micrographs demonstrating human corneal epithelial cells after storage in Optisol GS (A) and after Optisol GS + EBOC (B–D). Loss of superficial cells along with nuclear shrinkage, disorganized cytoplasm and membrane ruptures is seen after Optisol GS. Notice cubical basal cells and layers of horizontal superficial cell in (B–D) after Optisol GS + EBOC. Desmosomes (**) and hemidesmosomes (***) are seen in both systems. In addition, there are advanced foot processes (*) in the limbal area before and after the transfer to EBOC. S, superficial epithelial cells; B, basal cells; BM, basement membrane; EBOC, Eye Bank Organ Culture.

By TEM, a close to normal structure with regular chromatin, detectable mitochondria, foot processes and adhesion complexes were most evident in cells in the basal layers of the limbal area. Loss of normal cytoplasmic structure, nuclear deformation and membrane ruptures generally increased into superficial layers and into the more central corneal epithelium (Fig. 4A).

### Optisol GS storage + organ culture (group 2)

Samples were generally covered with 2–3 but occasionally up to five layers of epithelial cells. Cells in the superficial layers showed a dense staining for Occludin (Fig. 1), a varying density for E-cadherin in all layers, and only scattered cells were positive for Connexin-43 (Fig. 1). Cells positive for Ki67, p63, p63α, Vimentin and ABCG2 were seen in basal and suprabasal limbal and corneal layers (Fig. 2). In the limbal area, 7% of the cells were Ki67 positive, and the ratio between cells in the basal versus suprabasal layer was 11.1. Cell counts for cell number, Ki-67^+^, PCNA^+^ and p63^+^ cells after storage in Optisol GS and after subsequent storage in OC are shown in Table 2.

**Table 2:** Counting of positive cells after IHC staining with Ki-67, PCNA and p63, and the counting of cells with intact nucleus after H&E staining. *t*-test; spss output

Group name	Mean	SD	Significance (2-tailed)
PCNA			
Optisol GS	66.7	33.4	0.022
Optisol GS + OC	87.6	15.9	
Ki-67			
Optisol GS	7.2	7.7	0.077
Optisol GS + OC	18.3	23.3	
p63			
Optisol GS	77.1	12.5	0.857
Optisol GS + OC	77.9	14.6	
H&E			
Optisol GS	65.6	29.2	0.001
Optisol GS + OC	91.1	7.6	

Moderate staining for 8-OHdG and PCNA was widespread in the epithelium (Fig. 3), and semiquantitative evaluation of the former did not reveal any difference between samples after Optisol GS and after subsequent EBOC. An apparent overall increase in Caspase-3 staining was not detected. A slight positive reaction was observed in scattered cells with an apparent normal nuclear morphology. The TUNEL assay was positive in some cells with deformed nuclei (picture not shown).

Transmission electron microscopy generally showed an organized cytoplasm with detectable mitochondria, endoplasmic reticulum, intermediate filaments, a regular chromatin pattern, intercellular desmosomal complexes and basal hemidesmosomal attachments (Fig. 4B–D).

### Group 1 and group 2

#### RT-PCR

**Fig. 5:**
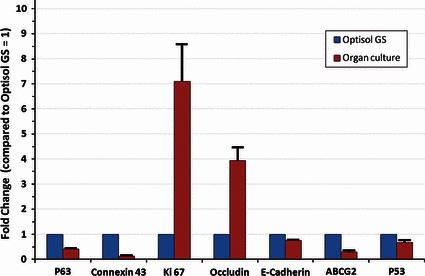
Real-time RT-PCR analysis for *p63*, *Connexin-43*, *Ki-67*, *Occludin*, *E-Cadherin, ABCG2* and P53 genes.

Results from gene expression analysis after Optisol GS and after Optisol GS + OC storage are presented in Fig. 5. In the latter group, there was a significant increase in the expression of the tight junction marker *Occludin*, while *Connexin-43* and *E-cadherin*, markers for gap and adherens junctions, respectively, were downregulated. As for proliferation, *Ki67* was upregulated, while the expression of putative progenitor/stemness-related genes *ABCG2* and *p63* was downregulated. P53 showed a reduction from 1 (SD 0.15) in Optisol GS to 0.4 (SD 0.03) after EBOC.

#### Lipid peroxidation and DNA damage

**Fig. 6:**
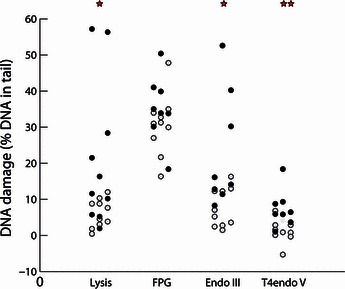
DNA damage in corneal epithelial cells before (grey symbols) and after (black symbols) transferring from Optisol to OC. Net enzyme-sensitive sites are shown. It was impossible to calculate accurately the enzyme-sensitive sites for two samples (after transfer) because the levels of strand breaks were too high. Student’s *t*-test was used to compare the amounts of different DNA lesions before and after transferring the tissue to the Eye Bank OC system (*p < 0.05, **p < 0.01).

Lipid peroxidation, assayed by measuring MDA, was detected after Optisol GS as well as after Optisol GS + OC and the concentration increased from 4.6 ± 2.1 mm/mg protein to 7.3 ± 0.9 mm/mg protein. In addition, the level of strand breaks was very low with a mean value of 5.7 ± 3.6% tail DNA in cold-stored tissue. Enzyme-sensitive sites were generally not much increased by organ culture, except for some samples that showed a substantial increase in EndoIII-sensitive sites (oxidized pyrimidines) that increased markedly in three of the 10 samples, while the levels of FPG-sensitive sites were similar in the two groups. The levels of T4endoVsites increased in all but one sample (Fig. 6).

## Discussion

The morphological alterations observed in the epithelium after storage in Optisol GS were similar to those described in previous studies, including a loss of superficial cell layers and occasional exposure of the basement membrane (Greenbaum et al. 2004). This deterioration explains the lack of staining for the tight junction protein Occludin in the samples. However, a primary storage in Optisol GS did not inhibit an increase in the gene expression and translation during subsequent organ culture.

We observed an intense immunostaining for E-cadherin and Connexin-43, markers detecting adherens and gap junctions, after Optisol GS. Translation, as well as the expression of these proteins, in particular that of Connexin-43, was reduced after organ culture. Varying expression of both E-cadherin and Connexin-43 has previously been described during wound healing and regeneration (Matic et al. 1997; Chang et al. 2008). Further, the expression of Connexin-43 also depends on the phenotype, a decreased level being observed in the basal limbal population (Hernandez Galindo et al. 2003). Our findings may therefore indicate an ongoing epithelial regeneration after 1 week in EBOC.

In epithelium on corneas stored in Optisol GS, subsequent organ culture was associated with an upregulation of proliferation marker *KI67*, and the density of cells positive for this marker indicated an ongoing regeneration. The transfer of nonwounded as well as wounded epithelium to EBOC has previously been shown to stimulate proliferative activity (Hjortdal et al. 1993; Kabosova et al. 2003; Chang et al. 2008), and Ki67-negative epithelium on EBOC corneas shows a transient proliferative increase when transferred to a complex medium commonly used to expand epithelium for transplant purposes (Joseph et al. 2004). In the latter study, density of cells positive for progenitor/stem cell marker p63 also showed a decrease after 2 weeks in culture. This is in line with our findings where we in addition show a decrease in the expression of progenitor/stem cell genes *p63* and *ABCG2*. Considering the potential use of Eye Bank donor tissue in various limbal epithelial transplant procedures (Pellegrini et al. 1997; Stenevi et al. 2002), the effect of commonly used Eye Bank storage systems on the preservation of ‘stemness’ deserves further investigation.

*Ex vivo* storage conditions are known to exert a continuous stress on the donor tissue. Eye Bank Organ Culture has been shown to affect the expression of Bcl2 and heat shock proteins involved in cellular defence against oxidative stress and apoptosis (Gain et al. 2001), and ‘cold’ storage is associated with an increase in nitrated proteins (Meisler et al. 2004).

Malondialdehyde, a potentially cytotoxic lipid peroxidation breakdown product, is known to accumulate in a range of disorders including bullous keratopathy, keratoconus, diabetic retinopathy and retinitis pigmentosa (Buddi et al. 2002; Johnsen-Soriano et al. 2008). DNA damage may act to destabilize cell function through an increased level of transcriptional errors (Ishibashi et al. 2005). Although some increase after EBOC was observed, we found a downregulation of p53, a sensor for cell and DNA damage and effector of cell cycle arrest and also of apoptosis. Cell death because of suprathreshold damage is seen with increasing time during ‘cold’ storage and also in EBOC (Komuro et al. 1999; Crewe & Armitage 2001). Such studies point at programmed cell death (apoptosis) as the main subtype. Caspase-3 is a commonly used marker for apoptosis although not specific, and the TUNEL reaction may in addition to apoptosis also be positive for necrotic cells. We could not detect any prominent increase in Caspase-3 immunostaining after subsequent EBOC of epithelium previously stored in a ‘cold’ system. However, the organ culture period in the present experiment was relatively brief. Also, a time-dependent detachment of apoptotic as well as necrotic cells from the epithelium may be facilitated by regenerative activity during EBOC.

In conclusion, the main purpose of our study was to investigate donor corneolimbal epithelium maintained in Optisol GS for alterations associated with a secondary incubation in a regular EBOC medium. After such a dual storage procedure, the epithelium demonstrated a layered and polarized morphology and a retained proliferative potential, although the expression of some genes related to differentiation and stemness was reduced. Some increase in oxidative stress was evident; however, there was a downregulation of the damage sensor P53 during EBOC. Our findings lend some support to the feasibility of EBOC pooling of donor tissue after storage in a ‘cold system’ with the benefits provided by such an option. There is an obvious need for further studies, in particular on the endothelium, in order to explore the potential and the safety of such a dual storage procedure.

## References

[b1] Armitage WJ, Easty DL (1997). Factors influencing the suitability of organ-cultured corneas for transplantation. Invest Ophthalmol Vis Sci.

[b2] Azqueta A, Shaposhnikov S, Collins AR (2009). DNA oxidation: investigating its key role in environmental mutagenesis with the comet assay. Mutat Res.

[b3] Borderie VM, Touzeau O, Bourcier T, Allouch C, Laroche L (2006). Graft reepithelialization after penetrating keratoplasty using organ-cultured donor tissue. Ophthalmology.

[b4] Buddi R, Lin B, Atilano SR, Zorapapel NC, Kenney MC, Brown DJ (2002). Evidence of oxidative stress in human corneal diseases. J Histochem Cytochem.

[b5] Camposampiero D, Tiso R, Zanetti E, Ruzza A, Bruni A, Ponzin D (2003). Improvement of human corneal endothelium in culture after prolonged hypothermic storage. Eur J Ophthalmol.

[b6] Chang CY, Green CR, McGhee CN, Sherwin T (2008). Acute wound healing in the human central corneal epithelium appears to be independent of limbal stem cell influence. Invest Ophthalmol Vis Sci.

[b7] Crewe JM, Armitage WJ (2001). Integrity of epithelium and endothelium in organ-cultured human corneas. Invest Ophthalmol Vis Sci.

[b8] Ehlers N, Hjortdal J, Nielsen K (2009). Corneal grafting and banking. Dev Ophthalmol.

[b9] Gain P, Thuret G, Chiquet C, Dumollard JM, Mosnier JF, Campos L (2001). In situ immunohistochemical study of Bcl-2 and heat shock proteins in human corneal endothelial cells during corneal storage. Br J Ophthalmol.

[b10] Greenbaum A, Hasany SM, Rootman D (2004). Optisol vs Dexsol as storage media for preservation of human corneal epithelium. Eye (Lond).

[b11] Hernandez Galindo EE, Theiss C, Steuhl KP, Meller D (2003). Gap junctional communication in microinjected human limbal and peripheral corneal epithelial cells cultured on intact amniotic membrane. Exp Eye Res.

[b12] Hjortdal JO, Haaskjold E, Sorensen KE, Bjerknes R (1993). Cell kinetics of normal and healing rat corneal epithelium during organ culture. Acta Ophthalmol (Copenh).

[b13] Hjortdal JO, Ehlers N, Erdmann L (1997). Topography of corneal grafts before and after penetrating keratoplasty. Acta Ophthalmol Scand.

[b14] Ishibashi T, Hayakawa H, Ito R, Miyazawa M, Yamagata Y, Sekiguchi M (2005). Mammalian enzymes for preventing transcriptional errors caused by oxidative damage. Nucleic Acids Res.

[b15] Jeng BH, Meisler DM, Hollyfield JG, Connor JT, Aulak KS, Stuehr DJ (2002). Nitric oxide generated by corneas in corneal storage media. Cornea.

[b16] Johnsen-Soriano S, Garcia-Pous M, Arnal E (2008). Early lipoic acid intake protects retina of diabetic mice. Free Radic Res.

[b17] Joseph A, Powell-Richards AO, Shanmuganathan VA, Dua HS (2004). Epithelial cell characteristics of cultured human limbal explants. Br J Ophthalmol.

[b18] Kabosova A, Kramerov AA, Aoki AM, Murphy G, Zieske JD, Ljubimov AV (2003). Human diabetic corneas preserve wound healing, basement membrane, integrin and MMP-10 differences from normal corneas in organ culture. Exp Eye Res.

[b19] Kolstad A (1979). Organ cultured donor material for penetrating corneal grafts. A preliminary report. Acta Ophthalmol (Copenh).

[b20] Komuro A, Hodge DO, Gores GJ, Bourne WM (1999). Cell death during corneal storage at 4 degrees C. Invest Ophthalmol Vis Sci.

[b21] Matic M, Petrov IN, Rosenfeld T, Wolosin JM (1997). Alterations in connexin expression and cell communication in healing corneal epithelium. Invest Ophthalmol Vis Sci.

[b22] Meisler DM, Koeck T, Connor JT, Aulak KS, Jeng BH, Hollyfield JG, Stuehr DJ, Shadrach KG (2004). Inhibition of nitric oxide synthesis in corneas in storage media. Exp Eye Res.

[b23] Nelson JD, Lange DB, Lindstrom RL, Doughman DJ, Hatchell DL (1984). McCarey-Kaufman (MK) organ culture and MK medium-shifted corneas. Arch Ophthalmol.

[b24] Pellegrini G, Traverso CE, Franzi AT, Zingirian M, Cancedda R, De LM (1997). Long-term restoration of damaged corneal surfaces with autologous cultivated corneal epithelium. Lancet.

[b25] Rijneveld WJ, Remeijer L, van RG, Beekhuis H, Pels E (2008). Prospective clinical evaluation of McCarey-Kaufman and organ culture cornea preservation media: 14-year follow-up. Cornea.

[b26] Rijneveld WJ, Wolff R, Volker-Dieben HJ, Pels E (2011). Validation of tissue quality parameters for donor corneas, designated for emergency cases: corneal graft survival. Acta Ophthalmol.

[b27] Slettedal JK, Lyberg T, Ramstad H, Beraki K, Nicolaissen B (2007). Regeneration of the epithelium in organ-cultured donor corneas with extended post-mortem time. Acta Ophthalmol Scand.

[b28] Stenevi U, Hanson C, Claesson M, Corneliusson E, Ek S (2002). Survival of transplanted human corneal stem cells. Case report. Acta Ophthalmol Scand.

